# Early exposure of infants to natural rotavirus infection: a review of studies with human rotavirus vaccine RIX4414

**DOI:** 10.1186/s12887-014-0295-2

**Published:** 2014-11-30

**Authors:** Nigel Cunliffe, Khalequ Zaman, Carlos Rodrigo, Serge Debrus, Bernd Benninghoff, Suryakiran Pemmaraju Venkata, Htay-Htay Han

**Affiliations:** University of Liverpool, Liverpool, England; ICDDR,B, Dhaka, Bangladesh; Germans Trias i Pujol University Hospital, Universidad Autónoma de barcelona, Barcelona, Spain; GlaxoSmithKline Vaccines, Wavre, Belgium; GSK Pharmaceuticals Pvt Ltd., Bangalore, India; GlaxoSmithKline Vaccines, 2301 Renaissance Boulevard, King of Prussia, PA 19406 U.S.A

**Keywords:** Rotavirus, Early protection, Gastroenteritis, Anti-rotavirus

## Abstract

**Background:**

Rotaviruses are the leading cause of severe acute gastroenteritis in children aged <5 years worldwide. A live attenuated human rotavirus vaccine, RIX4414 has been developed to reduce the global disease burden associated with rotavirus gastroenteritis. Serum anti-rotavirus immunoglobulin A (IgA) antibody measured in unvaccinated infants during clinical trials of RIX4414 reflects natural rotavirus exposure, and may inform the optimal timing for rotavirus vaccination.

**Methods:**

We reviewed phase II and III randomized, placebo-controlled clinical trials conducted by GlaxoSmithKline Vaccines, Wavre, Belgium between 2000 and 2008 which used the commercial formulation of RIX4414 lyophilized vaccine. We included trials for which demographic data and pre-dose-1 and post-last-dose anti-rotavirus IgA antibody status were available from placebo recipients.

**Results:**

Sixteen clinical trials met the inclusion criteria. The studies were conducted across Africa (N = 3), Asia (N = 4), Latin America (N = 4), Europe (N = 4) and North America (N = 1). Overall, 46,398 infants were enrolled and among these, 20,099 received placebo. The mean age at pre-dose-1 time point ranged from 6.4 − 12.2 weeks while the mean age at post-last-dose time point ranged from 13.5 − 19.6 weeks. The anti-RV IgA seropositivity rates at both time points were higher in less developed countries of Africa, Asia and Latin America (pre-dose-1: 2.1%-26.3%; post-last-dose: 6.3%-34.8%) when compared to more developed countries of Asia, Europe and North America (pre-dose-1: 0%-9.4%; post-last-dose: 0%-21.3%), indicating that rotavirus infections occurred at a younger age in these regions.

**Conclusion:**

Exposure to rotavirus infection occurred early in life among infants in most geographical settings, especially in developing countries. These data emphasize the importance of timely rotavirus vaccination within the Expanded Program on Immunization schedule to maximize protection.

## Background

Rotaviruses are a leading cause of severe acute gastroenteritis, resulting in approximately 453,000 annual deaths among children less than five years of age [1] with over 85% of these deaths occurring in the less developed countries of Asia and Africa [1,2]. Children typically experience multiple rotavirus infections during childhood, which may result in mild or asymptomatic infection to severe, life-threatening illness [3]. The first rotavirus infection is generally the most severe with subsequent rotavirus infections generally resulting in less severe disease outcomes because of acquisition of protective immunity, the extent of which varies by location [4,5].

Immunization of infants with oral, live attenuated rotavirus vaccine that mimics natural infection, prior to their first exposure to natural rotavirus infection is considered the best strategy to reduce the global disease burden [3,4]. A live attenuated human rotavirus vaccine, RIX4414 (*Rotarix*™, GlaxoSmithKline Vaccines, Wavre, Belgium) is administered orally according to a two dose schedule. The first dose can be administered as early as 6 weeks of age with a minimum of 4 weeks interval recommended between doses [6]. RIX4414 has undergone an extensive worldwide evaluation program. More than 30 clinical studies have been conducted to evaluate its safety, immunogenicity and efficacy, involving over 100,000 children in five continents. Such safety and efficacy studies in Europe [7], Latin America [8] and Asia [9] have confirmed that the vaccine is safe [10], well-tolerated [11] and efficacious (range: 80-96%) in preventing severe rotavirus gastroenteritis in the first two years of life. RIX4414 is now licensed in over 110 countries [12] and is included in the national immunization programs of low income/developing countries as well as in high income/developed countries.

From a public health perspective, it is important to identify the optimal age for the completion of rotavirus vaccination to obtain maximum benefit. To achieve this, we evaluated data obtained from placebo-controlled clinical trials conducted by GSK Biologicals using RIX4414 across different regions of the world. From all these studies, data on anti-rotavirus immunoglobulin A (IgA) antibody levels at pre-dose-1 and post-last-dose time points in the placebo recipients (of the total vaccinated cohort) were examined. The data available from the clinical trials reported in this review were used to assess the trend in exposure and age at infection.

## Methods

Clinical study reports of all randomized, double-blind and placebo-controlled phase II and phase III trials conducted between 2000 and 2008 using the commercial lyophilized formulation of RIX4414 vaccine were reviewed. Only studies with available data on anti-rotavirus IgA antibody seropositivity status at pre-dose-1 and post-last-dose time points for placebo recipients were included.

In all the included studies, each dose of the commercial formulation of RIX4414 contained at least 10^6.0^ median cell-culture infective doses (CCID_50_) of the vaccine strain. The placebo contained the same constituents as the active vaccine but without the virus component. Both were reconstituted with liquid calcium carbonate-based buffer before administration.

Blood samples were collected at pre-dose-1 and one to two months post-last-dose of placebo to measure the anti-rotavirus IgA antibody concentration using ELISA (Laboratory of Dr R. Ward, Children’s Hospital Medical Centre, Cincinnati, USA or at GlaxoSmithKline Laboratories, Rixensart, Belgium). The assay cut-off for seropositivity was set at 20 U/ml [13,14].

The demographic and serological data of the placebo group of the total vaccinated cohort were included in the analysis. The placebo group of the total vaccinated cohort comprised infants who had received at least one dose of placebo. Demography in terms of age range, gender and race were tabulated per study. Anti-rotavirus IgA seropositivity rates pre-dose-1 and one to two months post-last-dose of placebo and the mean age with standard deviation at the pre-dose-1 and post-last-dose time points were tabulated per study.

In all the study centers, the protocols, amendments and informed consent forms were reviewed and approved by the respective ethics committees. These studies were performed in accordance with the Good Clinical Practice guidelines and Declaration of Helsinki where applicable. Written informed consent was obtained from the parents/guardians of participating infants before carrying out any study-related procedures.

## Results

Of the 27 clinical study reports reviewed, 16 studies met the inclusion criteria (Table 1). Among the excluded studies, five studies had formulation of the vaccine with less than 10^6.0^ CCID_50_ of vaccine strain, one study had liquid formulation of RIX4414 vaccine, two studies did not include placebo groups and for the remaining three studies pre-dose-1 and/or post-last-dose anti-rotavirus IgA antibody data were unavailable. The included studies were conducted in Africa (N = 3), Asia (N = 4), Latin America (N = 4), Europe (N = 4) and North America (N = 1). Two studies (Rota-054 [11] and Rota-022 [15]) enrolled pre-term babies and HIV-positive infants respectively. A total of 46,398 infants were enrolled in these 16 studies, of which 20,099 infants had received at least one dose of placebo.

**Table 1:** **Summary of the studies included**

**Region**	**Countries**	**Study**	**Phase and design**	**Number of doses and Dosing schedule**	**Total number of enrolled infants**	**Number of infants in the placebo group**	**Reference**
**Africa**	South Africa	444563/013 (Rota-013)	Phase II, randomized, double-blind, placebo-controlled	2 or 3 doses; 0,1,2 month	475	96	**-**
	South Africa	444563/022 (Rota-022)	Phase II, randomized, double-blind, placebo-controlled	3 doses; 0,1,2 month	100	50	[15]
	South Africa, Malawi	102248 (Rota-037)	Phase III, randomized, double-blind, placebo-controlled	2 or 3 doses; 0,1,2 month	4939	1641	[16]
**Asia**	Korea	103478 (Rota-041)	Phase II, randomized, double-blind, placebo-controlled	2 doses; 0,2 month	161	52	[17]
	India	103792 (Rota-044)	Phase IIIb, randomized, double-blind, placebo-controlled	2 doses; 0,1 month	363	181	[18]
	Bangladesh	103992 (Rota-045)	Phase II, randomized, double-blind, placebo-controlled	2 doses; 0,1 month	300	98	[19]
	Singapore, Hong Kong, Taiwan	444563/028/029/030 (Rota-028, −029, −030)	Phase III, randomized, double-blind, placebo-controlled	2 doses; 0,1 or 2 month	10,708	5349	[9]
**Latin America**	Brazil, Mexico and Venezuela	444563/006 (Rota 006)	Phase IIb, randomized, double-blind and placebo-controlled trial	2 or 3 doses; 0,2 or 0,2,4 month schedule	2155	537	[20]
	Argentina, Brazil, Chile, Colombia, Dominican Republic, Honduras, Mexico, Nicaragua, Panama, Peru and Venezuela	444563/023 (Rota 023)	Phase III, randomized, double-blind, placebo-controlled	2 doses; 0,1-2 month	20,169	10,010	[21]
	Mexico, Colombia, Peru	444563/033 (Rota-033)	Phase II, randomized, double-blind, placebo-controlled	2 doses; 0,2 month	854	124	-
	Dominican Republic	106260 (Rota-052)	Phase IIIb, randomized, double-blind, placebo-controlled	2 doses; 0,2 month schedule	200	100	[22]
**Europe**	Finland	444563/003* (Rota-003)	Phase II, randomized, double-blind, placebo-controlled	2 doses; 0,2 month schedule	192	16	[23]
	Finland	104480 (Rota-048)	Phase II, randomized, double-blind, placebo-controlled	2 doses; 0,1 month	250	50	[12]
	Finland, Czech Republic, France, Germany, Italy, Spain	102247 (Rota-036)	Phase IIIb, randomized, double-blind, placebo-controlled	2 doses; 0,1-2 month schedule	3994	1348	[7]
	France, Portugal, Poland and Spain	106481 (Rota-054)	Phase IIIb, randomized, double-blind, placebo-controlled	2 doses; 0,1-2 month schedule	1009	339	[11]
**North America**	United States and Canada	444563/005** (Rota 005)	Phase II, randomized, double-blind, placebo-controlled	2 doses; 0,2 month schedule	529	108	[24]

*= Rota-003 was a dose escalation study with vaccines containing 10^5.3^, 10^5.6^ and 10^6.6^ CCID_50_ of RIX4414 strain. Here were are presenting the results of the placebo group whose corresponding vaccine contained 10^6.6^ CCID_50_ of RIX4414 strain.

**= Rota-005 used vaccines containing 10^5.6^ and 10^6.8^ CCID_50_ of RIX4414 strain. Here were are presenting the results of the placebo group whose corresponding vaccine contained 10^6.8^CCID_50_ of RIX4414 strain.

The demographic characteristics of the infants in all these studies are summarized in Table 2. The mean age at pre-dose-1 time point ranged from 6.4 weeks to 12.2 weeks while the mean age at post-last-dose time point ranged from 13.5 weeks to 19.6 weeks.

**Table 2:** **Demographic characteristics of the placebo group (of the Total vaccinated cohort)**

**Region**	**Study number**	**Age range of infants enrolled at the time of the first placebo dose (weeks)**	**Gender (%)**		**Majority race**
			**Male**	**Female**	
**Africa**	444563/013 (Rota-013)	5–10	53.1	46.9	African
	444563/022 (Rota-022)	5-10	50.0	50.0	African
	102248 (Rota-037)	2–11	51.2	48.8	African heritage/African American
**Asia**	103478 (Rota-041)	7–12	46.2	53.8	Korean
	103792 (Rota-044)	8–10	54.7	45.3	Indian
	103992 (Rota-045)	12–15	45.8	54.2	Bangladeshi
	444563 (Rota-028, −029, −030)	5 − 20	50.9	49.1	Chinese
**Latin America**	444563/006 (Rota 006)	6–12	50.3	49.7	Mestizo, Mestiza or Mixed
	444563/023 (Rota 023)	2–13	51.7	48.3	Hispanic
	444563/033 (Rota-033)	6–12	55.6	44.4	Hispanic
	106260 (Rota-052)	6 − 13	51.0	49.0	American Hispanic or Latino
**Europe**	444563/003 (Rota 003)	6-12	62.5	37.5	White
	104480 (Rota-048)	6–12	54.0	46.0	White - Caucasian/European heritage
	102247 (Rota-036)	5–18	51.3	48.7	White/Caucasian
	106481 (Rota-054)	5–14	50.7	49.3	White - Caucasian/European heritage
**North America**	444563/005 (Rota 005)	5-15	50.0	50.0	White/Caucasian

**Figure 1:**
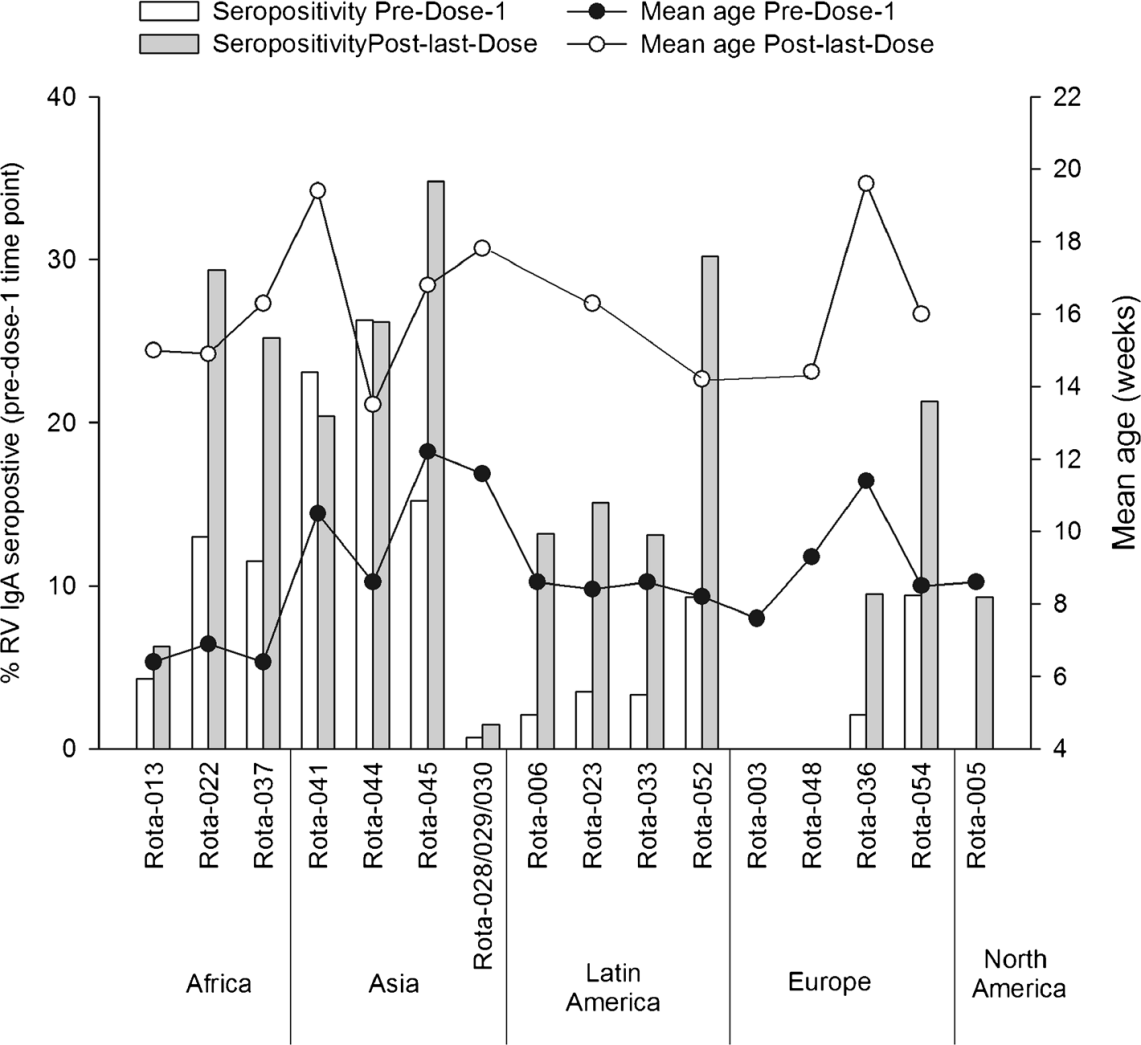
**Age and seropositivity rates at pre-dose-1 time point in the placebo groups (of the Total vaccinated cohort).** Numbers on top of the bars indicate mean age in weeks with standard deviation at pre-dose-1 time point.

**Africa:** Two phase II and one phase III study were conducted to evaluate the safety, efficacy and immunogenicity of RIX4414 (Rota-013, Rota-022 and Rota-037) [15,16]. At approximately 6 weeks of age (pre-dose-1 time point), the anti-rotavirus seropositivity rates in the placebo groups ranged from 4.3% (Rota-013) to 13.0% (Rota-022) (Table 3 and Figure 1). In the Rota-037 study conducted in South Africa and Malawi, the country- specific pre-dose-1 seropositivity rates were 12.2% (11/90) and 10.4% (7/67), respectively. In both Rota-013 and Rota-022, conducted in Africa, at the post-last-dose time point (15–16 weeks of age) the seropositivity rates increased to a maximum of 29.4% (Table 3). In Rota-037, although the overall anti-rotavirus seropositivity rate after the completion of the last placebo dose (8–21 weeks of age) was 25.2%, an apparent difference was observed in the seropositivity rates in South Africa (18.8% [172/917]) and Malawi (38.0% [176/463]).

**Table 3:** **Age and seropositivity rates at pre-dose-1 and post-last-dose time points in the placebo groups (of the Total vaccinated cohort)**

**Region**	**Countries**	**Study number**	**Mean age at pre-dose-1 (weeks) ± SD**	**Seropositivity rate at pre-dose-1**	**Mean age at last-dose of placebo (weeks) ± SD**	**Time between last-dose of placebo and post-last-dose blood draw (weeks)**	**Seropositivity rate post-last-dose**
							**% (n/N)**
				**% (n/N)**			
**Africa**	South Africa	444563/013 (Rota-013)	6.4 ± 1.07	4.3 (4/94)	15.0 ± 2.32	8	6.3 (5/80)
	South Africa	444563/022 (Rota-022)	6.9 ± 1.02	13.0 (6/46)	14.9 ± 1.64	8	29.4 (10/34)
	South Africa, Malawi	102248 (Rota-037)	6.4 ± 0.97	11.5 (18/157)	16.3 ± 1.51	4	25.2 (348/1380)
**Asia**	Korea	103478 (Rota-041)	10.5 ± 0.92	23.1 (12/52)	19.4 ± 1.09	8	20.4 (10/49)
	India	103792 (Rota-044)	8.6 ± 0.69	26.3 (45/171)	13.5 ± 1.12	4	26.2 (43/164)
	Bangladesh	103992 (Rota-045)	12.2 ± 0.47	15.2 (14/92)	16.8 ± 0.52	4	34.8 (32/92)
	Singapore, Hong Kong, Taiwan	444563 (Rota-028, −029, −030)	11.6 ± 2.37	0.7 (1/135)	17.8 ± 1.55	4–8	1.5 (2/132)
**Latin America**	Brazil, Mexico and Venezuela	444563/006 (Rota 006)	8.6 ± 1.98	2.1 (11/528)	NA*	8	13.2 (24/182)
	Argentina, Brazil, Chile, Colombia, Dominican Republic, Honduras, Mexico, Nicaragua, Panama, Peru and Venezuela	444563/023 (Rota 023)	8.4 ± 2.37	3.5 (15/432)	16.3 ± 3.77	4–8	15.1 (60/398)
	Mexico, Colombia, Peru	444563/033 (Rota-033)	8.6 ± 2.20	3.3 (4/121)	NA*	8	13.1 (14/107)
	Dominican Republic	106260 (Rota-052)	8.2 ± 1.80	9.3 (9/97)	14.2 ± 1.83	6	30.2 (29/96)
**Europe**	Finland	444563/003 (Rota 003)	7.6 ± 1.75	0.0 (0/16)	NA*	4	0.0 (0/14)
	Finland	104480 (Rota-048)	9.3 ± 2.04	0.0 (0/49)	14.4 ± 2.12	4	0.0 (0/48)
	Finland, Czech Republic, France, Germany, Italy, Spain	102247 (Rota-036)	11.4 ± 1.84	2.1 (10/479)	19.6 ± 2.74	12	9.5 (45/473)
	France, Portugal, Poland and Spain	106481 (Rota-054)	8.5 ± 1.78	9.4 (9/96)	16.0 ± 2.95	4	21.3 (20/94)
**North America**	United States and Canada	444563/005 (Rota 005)	8.6 ± 1.31	0.0 (0/95)	NA*	8	9.3 (8/86)

SD = Standard deviation.

n = number of infants in placebo groups with anti-rotavirus IgA antibody concentration ≥20 U/ml.

N = number of infants in placebo groups with available results.

Note: In all the studies mentioned above, the assessment of seropositivity rates was performed on a subset of total number of enrolled infants.

NA* = No age data calculated for the post-last-dose time point.

**Asia:** Four studies, one each in India (Rota-044), Bangladesh (Rota-045), Korea (Rota-041) and a combined study in Singapore, Hong Kong and Taiwan (Rota-028, −029, −030) evaluated the safety, efficacy and immunogenicity of the RIX4414 vaccine [9,18,19]. In this region, in the less developed countries, a maximum seropositivity rate of 26% was observed at pre-dose-1 time point (approximately 9–12 weeks of age) in India (Table 3 and Figure 1). The seropositivity rate rose to a maximum of 35% at approximately 18 weeks of age in Bangladesh (Table 3). In the developed countries, the seropositivity rates at pre-dose-1 and post-last-dose time points were 0.7% and 1.5%, respectively (Figure 1).

**Latin America:** In the immunogenicity and safety studies conducted in Latin America (Rota-006, Rota-023, Rota-033 and Rota-052) [20-22] the anti-rotavirus IgA antibody seropositivity rates were close to a maximum of 9% (~8 weeks of age) at pre-dose-1 and 30% (~16 weeks of age) at post-last-dose time points (Table 3). The post-last-dose seropositivity rates were very similar to that seen in Asia.

**Europe:** In Rota-003, Rota-048 [12], Rota-036 [25] and Rota-054 [11], the pre-dose-1 anti-rotavirus seropositivity rates ranged from 0% to 9% (9–11 weeks of age). During the post-last-dose time point (16–20 weeks of age), the seropositivity rates rose to a maximum of 21% (Table 3 and Figure 1).

**North America:** In a phase II study conducted in United States and Canada (Rota-005), none of the infants were seropositive at the pre-dose-1 time point (~9 weeks of age). The seropositivity rate rose to 9.3% at post-last-dose time point (Table 4).

**Table 4:** **Trial registration numbers**

**Country**	**Study number/NCT number**
Africa	Rota-013 (444563/013)/NCT00383903
	Rota-022 (444563/022)/NCT00263666
	Rota-037 (102248)/NCT00241644
Asia	Rota-028, −029, −030 (444563/028-029-030)/NCT00197210
	Rota-041 (103478)/NCT00134732
	Rota-044 (103792)/NCT00289172
	Rota-045 (103992)/NCT00139334
Latin America	Rota-006 (444563/006)/NCT00385320
	Rota-023 (444563/023)/NCT00140673
	Rota-033 (444563/033)/NCT00757770
	Rota-052 (106260)/NCT00396630
Europe	Rota-003 (444563/003)
	Rota-036 (102247)/NCT00140686
	Rota-048 (104480)/NCT00137930
	Rota-054 (106481)/NCT00420745
North America	Rota-005 (444563/005)/NCT00729001

## Discussion

Assessment of anti-rotavirus IgA seropositivity rate in the placebo groups at pre-dose-1 and post-last-dose time points in 16 studies across five geographical regions has provided information on the approximate age at which children are naturally infected by rotaviruses.

Although, epidemiological studies from different regions have shown that the incidence of rotavirus infection is highest in children aged 6–23 months, it is recognized that rotavirus infection may occur in neonates and children aged less than 2 months [26,27]. A study conducted in 11 Latin American countries indicated that up to 11% of rotavirus gastroenteritis (RV GE) cases were observed in children younger than 3 months [28]. The REVEAL study conducted across seven European countries showed that the percentage of RV GE in the 0–2 months age group ranged from 0.8% in Sweden to 6.1% in France [27,29]. An hospital-based study in Malawi demonstrated that 7.6% of severe RV GE cases occurred in infants below three months of age [30]. A previous report indicated that rotavirus infections in neonates are mostly nosocomial and typically asymptomatic [31].

In line with this, the present review also showed that infants were at risk of becoming infected with rotaviruses prior to RV vaccination, as demonstrated by the presence of anti-rotavirus IgA seropositivity rate at pre-dose-1 time point in most of the regions. At pre-dose-1 and post-last-dose time points, highest seropositivity rates (26% and 34% at pre-dose-1 and post-last-dose) were observed at a younger age in less developed countries of Asia followed by Africa and Latin America. The maximum baseline seropositivity rate was 26% in India (Rota-044), which is in line with previously published data [5], indicating that natural rotavirus infections may occur very early in life furthering the need for neonatal immunization. However, the seropositivity rates observed in high-income Asian countries (Rota-028, −029, −030) at post-last-dose time point was not only lower (1.5%) than that observed in other low-income Asian countries, but was in fact lower than that observed in Europe and North America. This suggests that socioeconomic conditions, overcrowding, malnourishment, sanitation and personal hygiene or other factors could expose children in less developed countries at high risk of exposure to rotavirus at a younger age compared to children living in developed or high-income countries [3,32]. Furthermore, the Asian Rotavirus Surveillance Network data indicated that rotavirus disease-associated hospitalizations occur more frequently at a younger age in low income than in high income countries [33].

In Africa the anti-rotavirus seropositivity rate after the last placebo dose was greater in Malawi compared to South Africa suggesting a higher exposure to wild-type rotavirus in the first five months of life in Malawian than of South African infants. This observation may partly be explained by the different enrolment patterns employed during the Phase III trial (Rota-037) in these countries. In South Africa, enrolment was timed before the rotavirus season while in Malawi, enrolment was done all year-round and no clear seasonality was observed [16]. In addition, lack of seasonality itself may be one of the reasons for increased rotavirus infection occurring at an earlier age in tropical countries where children are exposed to rotavirus all year-round [34,35]. Furthermore, a previously conducted study in Venezuela reported that the infection rate and severity of the disease increased in environments with minimal seasonality [36].

Although the overall seropositivity rates at pre-dose-1 and post-last-dose were lowest in Europe, a wide disparity was observed between studies. In the Rota-054 study, the seropositivity rates at both time points were similar to that observed in Asia, Africa and Latin America. Such a difference may be attributed to the premature condition of the study population, making the infants susceptible to rotavirus disease at an earlier age [11].

There are some limitations to this review: firstly, the enrolment age across all the included studies was different. Therefore, it was not possible to estimate the actual age at which infants were first infected with rotavirus and the severity of possible clinical symptoms. Secondly, the seropositivity data available for each study were independent in terms of age-limit at the time of dose-1 and during post-last-dose time point, hence the age groups of infants were not uniform. Furthermore, the lack of seropositivity observed at pre-dose-1 and post-last-dose time points in the Rota-003 and −048 trials could be partly due to the low number of subjects in each trial (n = 16 and n = 50, respectively). Thirdly, since all these studies were conducted under a clinical trial setting with definite criteria for enrollment, the data may not precisely reflect a real-life setting. Additionally, there are some studies that have shown that maternal IgA antibody move transplacentally at a slow rate [37,38] and the presence of RV-specific IgA in infant sera at a young age could be due to the presence of maternal antibodies. Finally, it has also been suggested in early reports that maternal antibodies play a role in modulating the immunogenicity and efficacy of rotavirus vaccines [39]; therefore the timing of vaccination needs to be carefully selected. However, studies have also demonstrated the administering the first dose of the rotavirus vaccine in the neonatal period proves efficacious and affords protection early on in life [40].

## Conclusion

Exposure to rotavirus infection is common in the first six months of life and varies by geographic region with infants in some less developed settings having higher rates of early RV infection as compared to that of infants in developed settings. These observations reinforce the need for completion of rotavirus vaccination in a timely fashion when delivered through childhood immunization programs.

## Abbreviations

IgA: Immunoglobulin A
CCID_50_: Cell-culture infective doses
RV GE: Rotavirus gastroenteritis
